# AI-Aquatica-RS: A Modular Python Framework for Reproducible Fusion of Remote-Sensing-Derived Spectral Indices and In Situ Water-Quality Observations

**DOI:** 10.3390/s26165162

**Published:** 2026-08-14

**Authors:** Tymoteusz Miller, Irmina Durlik

**Affiliations:** 1 Institute of Marine and Environmental Sciences, University of Szczecin, 70-364 Szczecin, Poland; 2 Faculty of Data Science and Information, INTI International University, Nilai 71800, Negeri Sembilan, Malaysia; 3 Faculty of Navigation, Maritime University of Szczecin, 70-500 Szczecin, Poland; i.durlik@pm.szczecin.pl

**Keywords:** remote sensing, in situ observations, water quality, spectral indices, temporal alignment, machine learning, data fusion, reproducible software, synthetic benchmark

## Abstract

Remote-sensing-derived spectral indices and in situ measurements provide complementary information for aquatic monitoring, but their practical integration is complicated by asynchronous observations, heterogeneous tables, missing acquisitions, and non-reproducible preprocessing. This study presents AI-Aquatica-RS, a modular Python framework for spectral-index calculation, station-aware nearest-neighbor temporal alignment, feature-set construction, regression benchmarking, command-line execution, and structured result export. The software was evaluated using a fully synthetic controlled benchmark; no real satellite scenes or field-monitoring measurements were used. The benchmark comprised 1080 daily in situ-like observations from six stations and 181 unique remote-sensing-like acquisitions generated as water-like surface-reflectance proxies. A ±3-day alignment tolerance produced a shared complete-case cohort of 954 records. To ensure a fair comparison, the in situ-only, spectral-index-only, and fused configurations were evaluated on exactly the same 667 training and 287 validation records. The fused configuration achieved the best performance using ridge regression (RMSE = 3.481 NTU, MAE = 2.768 NTU, R^2^ = 0.729), compared with RMSE values of 5.086 NTU for the in situ-only configuration, and 5.538 NTU for the spectral-index-only configuration. The benchmark demonstrates reproducible execution and recovery of complementary information under controlled conditions; it does not constitute environmental validation. AI-Aquatica-RS provides an extensible software layer for future studies using real satellite products, monitoring networks, sensor-specific preprocessing, and spatially blocked validation.

## 1. Introduction

Aquatic monitoring increasingly relies on heterogeneous observations collected at different spatial and temporal scales. In situ measurements provide direct physicochemical information, including temperature, conductivity, pH, dissolved oxygen, nutrients, chlorophyll-related proxies, suspended matter, and turbidity. These observations are locally interpretable but often sparse, irregular, and labor-intensive [1,2]. Remote sensing supplies repeated spatial coverage and spectral information associated with water extent, suspended matter, phytoplankton-related signals, colored dissolved organic matter, and surface optical variability [3,4,5,6,7,8]. However, satellite-derived variables remain indirect and are affected by atmospheric correction, clouds, sun glint, adjacency effects, mixed pixels, water depth, and sensor-specific spectral response [9,10,11,12,13,14,15,16,17,18].

The two observation types are therefore complementary rather than interchangeable. Field observations can calibrate and interpret optical signals, whereas remote-sensing-derived features can add spatial and temporal context to point-based sampling [5,6,17]. This complementarity is particularly relevant to machine-learning-based reconstruction, in which an unobserved or difficult-to-measure water-quality variable is estimated from available covariates. Before such a model can be evaluated, the workflow must standardize dates and station identifiers, calculate spectral indices, align asynchronous records, construct alternative feature sets, apply one reproducible evaluation protocol, and export auditable predictions and metrics [18,19,20,21,22,23].

Many studies implement these steps in project-specific notebooks or scripts. Consequently, comparisons may be affected by undocumented temporal tolerances, different complete-case subsets, inconsistent train–test splits, or manually assembled outputs. Reproducible environmental AI requires the data-engineering decisions to be treated as part of the method rather than as an informal preliminary step [24,25,26,27,28].

This study introduces AI-Aquatica-RS, a modular Python framework for the tabular integration stage that follows satellite-image preprocessing. It assumes that station-level spectral bands or derived features are available and provides reusable components for validation, normalized-difference index calculation, station-aware temporal alignment, feature-set construction, regression benchmarking, and structured export. It does not perform satellite acquisition, atmospheric correction, cloud masking, water-body segmentation, or pixel extraction.

The article makes four contributions. First, it provides a tested software architecture connecting in situ tables and remote-sensing-derived tables in a configuration-driven workflow. Second, it implements explicit nearest-neighbor alignment with traceability fields describing the matched acquisition and temporal difference. Third, it enforces a shared matched cohort and identical temporal split when comparing in situ-only, spectral-index-only, and fused feature configurations. Fourth, it provides a transparent controlled benchmark with example inputs, descriptive statistics, figures, predictions, and an output manifest.

The demonstration is fully synthetic. It is designed to verify software execution and determine whether the workflow can recover deliberately complementary information under controlled conditions. It is not presented as validation against real satellite scenes, real field measurements, or any specific Landsat or Sentinel-2 product.

## 2. Related Work

### 2.1. Remote Sensing and Aquatic Water-Quality Variables

Remote sensing is widely used to investigate optically active aquatic variables, including chlorophyll-a, turbidity, total suspended solids, colored dissolved organic matter, surface algal blooms, and water extent [3,7,8]. Multispectral missions such as Landsat and Sentinel-2 have increased access to visible, near-infrared (NIR), and shortwave-infrared (SWIR) observations [10,11,17]. Variables without a direct optical response, including nutrients, conductivity, dissolved oxygen, or biochemical oxygen demand, generally require indirect modeling and in situ calibration [9].

Normalized-difference transformations provide compact representations of band relationships. The Normalized Difference Vegetation Index (NDVI), Normalized Difference Water Index (NDWI), Modified Normalized Difference Water Index (MNDWI), and Normalized Difference Turbidity Index (NDTI) are commonly used as interpretable features, although their meaning depends on sensor, water type, atmospheric processing, and scene context [12,13,14,29,30,31,32,33,34,35]. Water pixels are generally characterized by relatively low NIR and SWIR reflectance, while turbid or vegetation-influenced conditions can increase red or NIR signals [4,17,36].

### 2.2. Machine Learning for Water-Quality Reconstruction

Linear regression, regularized regression, nearest-neighbor models, decision trees, random forests, extra trees, and boosting methods are suitable for moderate-sized environmental tables and can represent nonlinear interactions between optical and physicochemical predictors [19,20,21,22,23,35,37]. Their performance depends not only on model class but also on temporal correspondence, feature availability, sample definition, and validation design. Data fusion should therefore be tested against single-source baselines on the same observations rather than assumed to be beneficial.

### 2.3. Reproducible Environmental Software Workflows

Environmental workflows include ingestion, validation, quality control, transformation, temporal aggregation, feature engineering, model fitting, and export. Packages such as SaQC illustrate the value of explicit, traceable preprocessing components [24]. FAIR and machine-learning reproducibility principles further emphasize documented dependencies, controlled random seeds, reusable configurations, and machine-readable outputs [25,26,27,28]. AI-Aquatica-RS applies these principles specifically to the downstream fusion of station-level in situ and remote-sensing-derived tables.

### 2.4. Research Gap

Existing work often concentrates on sensor-specific retrieval algorithms or individual case studies. A practical gap remains for a lightweight software layer that explicitly connects spectral-index calculation, station-aware temporal alignment, fair feature-set comparison, and result export. AI-Aquatica-RS addresses this integration gap without attempting to replace established geospatial platforms such as Google Earth Engine, SNAP, QGIS, or sensor-specific atmospheric-correction workflows [32,33,38].

## 3. AI-Aquatica-RS Framework

### 3.1. Architecture and Processing Layers

**Figure 1:**
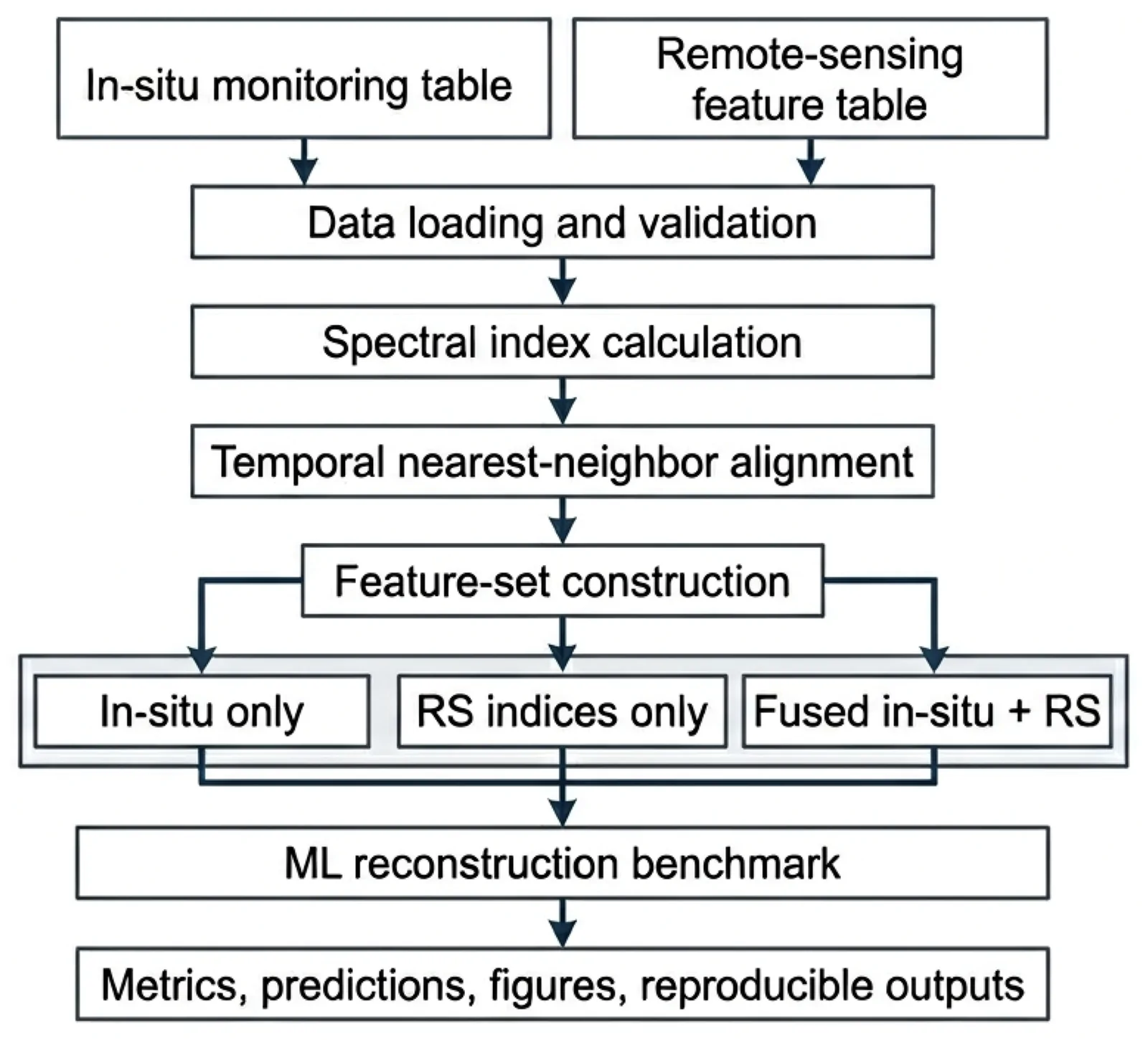
Conceptual workflow of AI-Aquatica-RS. In situ and remote-sensing-derived tables are validated, spectral indices are calculated, observations are aligned by station and date, and three feature configurations are evaluated using a common modeling cohort and common temporal split.

AI-Aquatica-RS follows a modular pipeline architecture with six processing layers: (1) tabular data input and schema validation; (2) spectral-index generation; (3) exact or nearest-neighbor temporal alignment by station; (4) feature engineering and construction of alternative predictor sets; (5) deterministic regression benchmarking; and (6) structured export of metrics, predictions, summaries, samples, and figures. Each layer can be used independently, while the complete benchmark can be executed through one configuration file (see Table 1 and Figure 1).

### 3.2. Configuration-Driven Execution

The complete demonstration is defined in configs/article_benchmark.yaml. The configuration records the simulation period, station count, revisit interval, missing-acquisition probability, temporal tolerance grid, selected tolerance, temporal cutoff, feature lists, model registry, random state, and output directories. The public command-line interface executes both validation and the complete workflow:

ai-aquatica-rs validate-fusion-config --config configs/article_benchmark.yaml
ai-aquatica-rs run-fusion-benchmark --config configs/article_benchmark.yaml

The legacy train-reconstruction command remains available for users who already possess a prepared single-table modeling dataset. The complete run–fusion–benchmark command additionally performs synthetic input generation, index calculation, temporal alignment, shared-cohort construction, feature–set comparison, and manuscript–output generation.

### 3.3. Scope

The framework begins at the tabular integration stage. Real applications must independently document satellite source, product level, atmospheric correction, cloud and shadow filtering, spatial extraction footprint, adjacency handling, and pixel aggregation. These upstream operations are deliberately outside the present software scope because they are sensor- and study-specific.

## 4. Materials and Methods

### 4.1. Controlled Benchmark Design

The benchmark was constructed to test the software under a deterministic multi-station scenario. Six stations were simulated for 180 consecutive days beginning on 1 January 2024, producing 1080 daily in situ-like observations. Remote-sensing-like acquisition opportunities occurred every five days. Each nominal acquisition was independently removed with a probability of 0.18 to represent unusable observations caused by cloud or quality filtering. A separate deterministic random-number stream was used for acquisition availability. With random state 42, the final table contained 181 unique remote-sensing-like acquisitions.

The synthesized in situ variables were water temperature (°C), conductivity (µS/cm), pH, 24 h rainfall (mm), and turbidity (NTU). Rainfall was zero with probability 0.69; otherwise, it followed a gamma distribution with shape 1.25 and scale 3.2. Antecedent rainfall was represented by a recursively decaying proxy. Temperature and pH included seasonal components and Gaussian noise, whereas conductivity additionally included station, cyclic, and antecedent-rainfall effects. The station effect was distributed evenly between −0.28 and 0.28 (see Table 2).

### 4.2. Latent Optical States and Water-like Band Proxies

Two latent states were generated: suspended-matter state S and algal-influence state A. Both were constrained to the interval [0, 1] by a logistic transformation and evolved through autoregressive recursions. The suspended state responded to antecedent rainfall, station effect, and a 72-day cyclic component; the algal state responded primarily to seasonality, station effect, and rainfall. Autoregressive coefficients of 0.78 and 0.84 were used for the suspended and algal logits, respectively, to ensure that optical conditions changed gradually enough for a multi-day matching experiment.

Red, green, NIR, and SWIR values were generated as dimensionless surface-reflectance proxies. The coefficients were selected so that NIR and SWIR were generally lower than visible reflectance, consistent with the qualitative behavior of open-water pixels, while elevated suspended or algal states increased visible and, to a lesser extent, NIR reflectance [4,17,36]. The values do not reproduce a particular sensor response or atmospheric-correction product.

G = clip(0.028 + 0.040S + 0.035A + εG, 0.003, 0.18)(1a)

R = clip(0.014 + 0.070S + 0.010A + εR, 0.002, 0.18)(1b)

NIR = clip(0.004 + 0.032S + 0.016A + εNIR, 0.001, 0.12)(1c)

SWIR = clip(0.0015 + 0.012S + 0.004A + εSWIR, 0.0005, 0.06)(1d)

**Figure 2:**
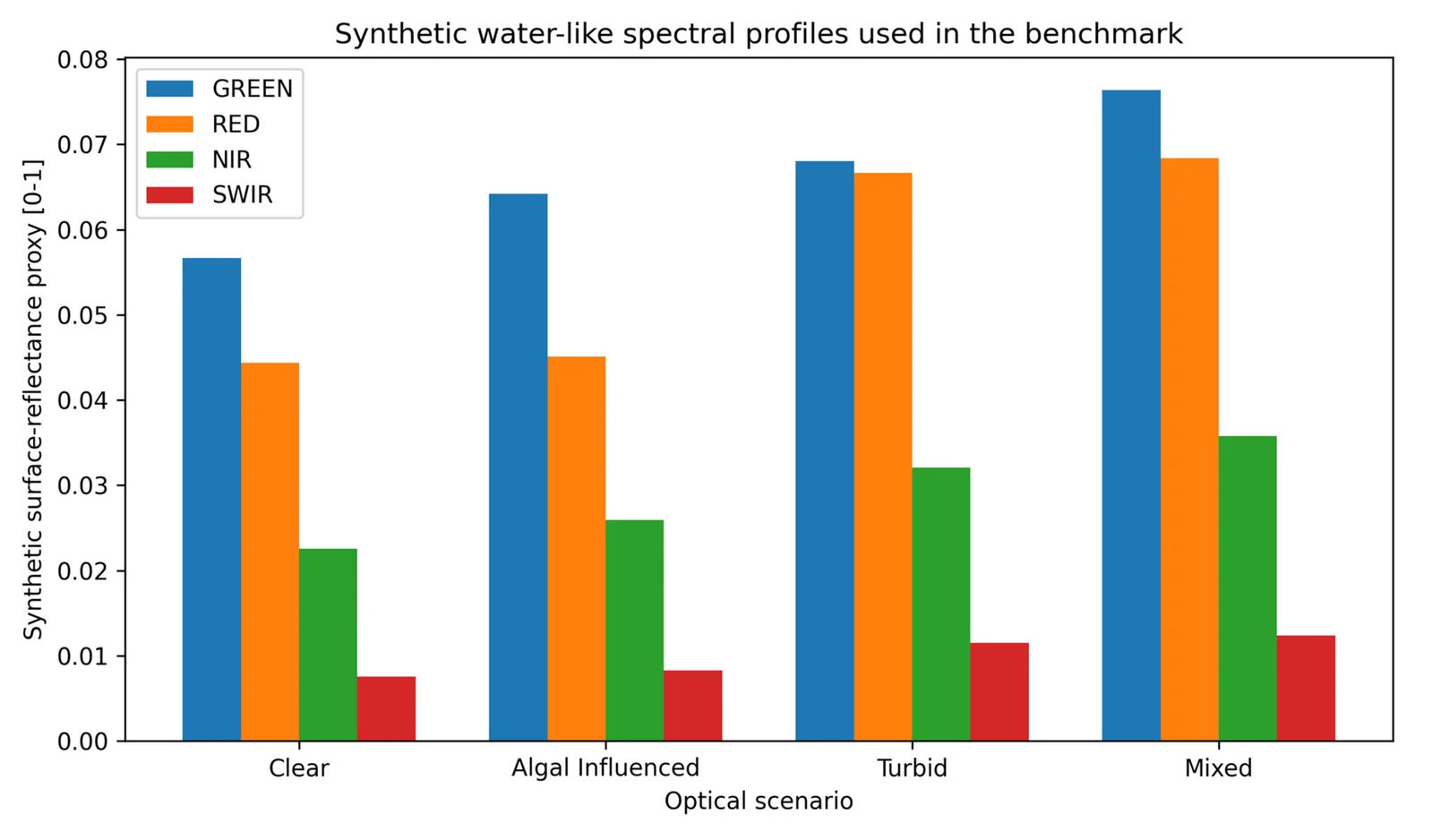
Mean synthetic surface-reflectance proxies by optical scenario. The profiles were designed to preserve water-like qualitative ordering, especially reduced NIR and SWIR relative to visible reflectance. They are synthetic and should not be interpreted as observations from a specific satellite sensor.

The independent Gaussian standard deviations were 0.0022, 0.0020, 0.0015, and 0.0008 for green, red, NIR, and SWIR, respectively. Each acquisition was assigned one descriptive scenario label—clear, turbid, algal-influenced, or mixed—using thresholds of 0.58 for the latent states. These labels are explanatory outputs and were not used as model predictors (Figure 2).

### 4.3. Synthetic Target Variable

The reconstruction target was explicitly defined as synthetic turbidity in nephelometric turbidity units (NTU). It was generated from the latent suspended-matter state, current rainfall, conductivity, pH, station effect, seasonal component, and independent Gaussian noise. Importantly, turbidity was not calculated directly from NDVI, NDWI, MNDWI, or NDTI. Spectral indices remained informative because the band proxies and turbidity shared the same latent suspended-matter process.

Turbidity = max[0.5, 3 + 30S + 0.45P + 0.055(C − 475) + 7(pH − 7.50) + 4E_s_ + 2Q + εᵧ], εᵧ ~ N(0, 1.60^2^) (2)

Here, P is 24 h rainfall, C is conductivity, E_s_ is the station effect, Q is the seasonal sine component, and S is the latent suspended-matter state. This construction creates controlled complementarity: the in situ predictors describe hydrometeorological, chemical, seasonal, and station context, while the optical indices provide an indirect observation of the latent suspended state (see Table 3 and Table 4).

Complete examples are exported as sample_synthetic_insitu.csv, sample_synthetic_remote_sensing.csv, and sample_aligned_modeling_cohort.csv. These files allow readers to inspect the exact table structure used by the framework without relying on screenshots.

### 4.4. Spectral-Index Calculation

The software calculates normalized-difference indices using one stabilized implementation:

ND(B_1_,B_2_) = (B_1_ − B_2_)/(B_1_ + B_2_ + ε) (3)

where ε prevents division by zero. NDVI uses NIR and red; NDWI uses green and NIR; MNDWI uses green and SWIR; and NDTI uses red and green [13,29,30,31]. The indices are computed once at the acquisition level before temporal alignment. Consequently, acquisition-level descriptive statistics contain 181 observations, whereas the aligned modeling table contains repeated references to acquisitions when one remote-sensing record is the nearest valid match for multiple daily in situ observations.

### 4.5. Temporal Alignment and Common Modeling Cohort

For each in situ observation at station s and date t, the nearest remote-sensing acquisition from the same station was selected if its absolute temporal difference did not exceed tolerance Δ:

AΔ(s,t) = arg minτ ∈ TR(s) |τ − t|, subject to |τ − t| ≤ Δ(4)

The benchmark evaluated Δ = 0, 1, 3, 5, and 7 days. The aligned table records the original in situ row identifier, matched acquisition identifier, matched acquisition date, match type, and signed time difference. The primary reconstruction experiment used Δ = 3 days.

A single common complete-case cohort was constructed after alignment. A row was retained only when the target and the union of all predictors required by the three feature configurations were available. This procedure produced 954 matched records. All configurations were then split chronologically at 1 May 2024, yielding exactly 667 training records and 287 validation records for every configuration. No configuration was allowed to use additional unmatched observations.

### 4.6. Feature Configurations and Models

The in situ-only configuration used water temperature, conductivity, pH, rainfall, sine and cosine day-of-year terms, and station indicators. The spectral-index-only configuration used NDVI, NDWI, MNDWI, NDTI, absolute temporal difference, and station indicators. The fused configuration combined both groups. Station indicators were derived from the six synthetic station identifiers.

The benchmark included median and mean baselines, ordinary linear regression, ridge regression, k-nearest neighbors, a decision tree, random forest, extra trees, gradient boosting, and histogram gradient boosting. All stochastic estimators used random state 42. Optional third-party boosting libraries were not required for the article benchmark.

### 4.7. Evaluation Metrics

Performance was evaluated on the common chronological validation subset using RMSE, MAE, R^2^, and MAPE [34].

RMSE = √[(1/n) Σ_i_(y_i_ − ŷ_i_)^2^] (5)

MAE = (1/n) Σ_i_|y_i_ − ŷ_i_| (6)

R^2^ = 1 − [Σ_i_(y_i_ − ŷ_i_)^2^/Σ_i_(y_i_ − ȳ)^2^] (7)

MAPE = (100/n) Σ_i_ |(y_i_ − ŷ_i_)/y_i_| (8)

RMSE and MAE are reported in NTU. The best model within each feature configuration was selected by minimum validation RMSE. The full model table was exported rather than reproduced in full in the manuscript.

### 4.8. Reproducibility Outputs

The package also exports four publication-ready figures and the complete synthetic input tables. The output manifest separates unique acquisition-level summaries from post-alignment modeling outputs, preventing ambiguity in record counts (see Table 5).

## 5. Results

### 5.1. Synthetic In Situ Variable Characteristics

The synthetic turbidity target ranged from 3.160 to 45.598 NTU, with a mean of 22.002 NTU and standard deviation of 7.107 NTU. The distributions therefore covered low-to-moderately elevated synthetic turbidity while remaining strictly positive for percentage-based metrics (see Table 6).

### 5.2. Temporal Alignment Coverage

**Figure 3:**
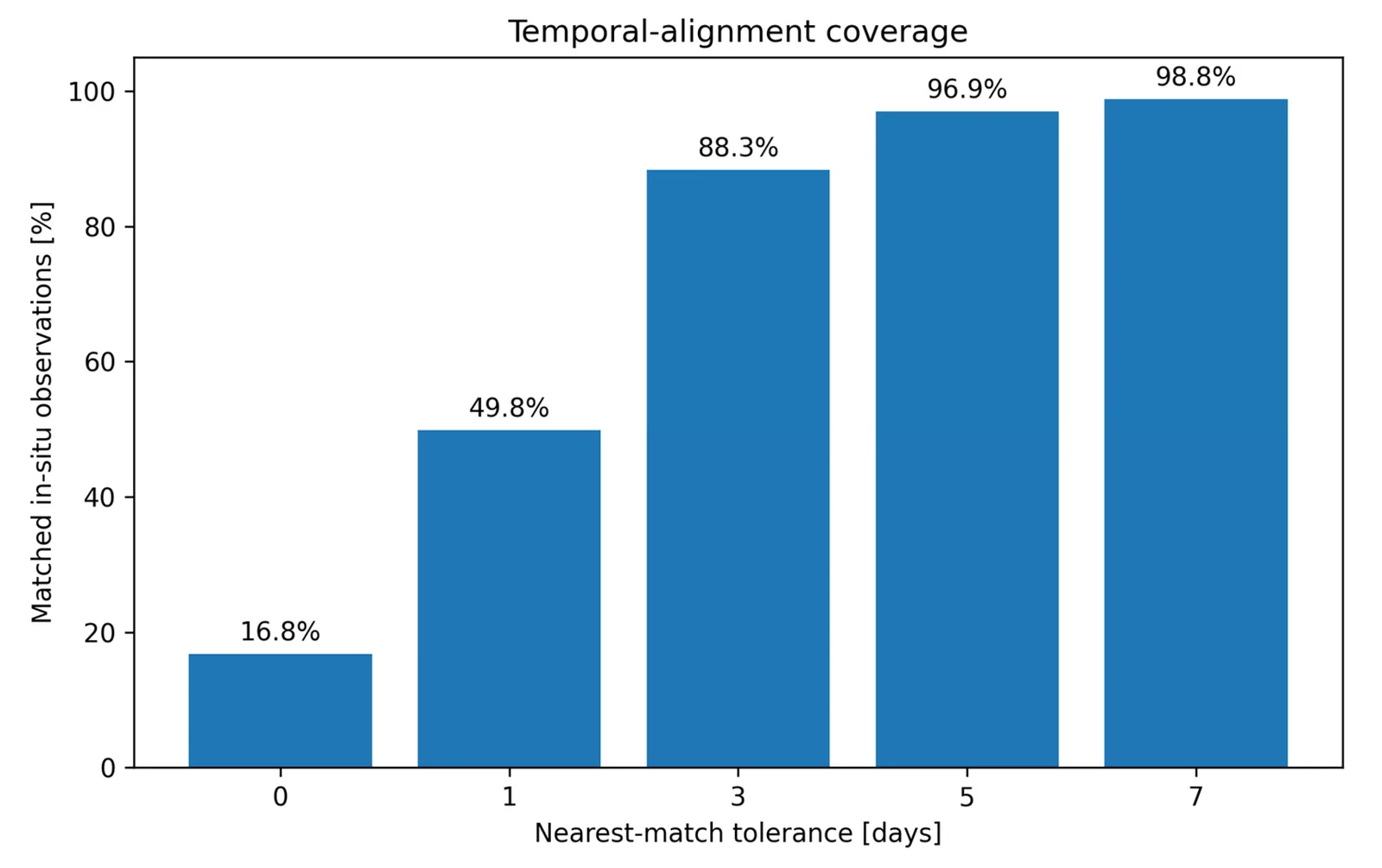
Temporal alignment coverage for the five evaluated tolerance values. Tolerance is a categorical experimental setting and is therefore displayed as a bar chart.

Exact-date matching retained 181 records (16.76%). A one-day tolerance increased coverage to 538 records (49.81%), and the selected three-day tolerance yielded 954 records (88.33%) with a mean absolute temporal difference of 1.31 days. Wider windows produced 1047 and 1067 matched records for five and seven days, respectively, but also increased the mean temporal difference (see Figure 3 and Table 7).

### 5.3. Acquisition-Level Spectral Characteristics

All acquisition-level counts equal 181 because these statistics were calculated before temporal alignment. The mean green, red, NIR, and SWIR proxies were 0.0619, 0.0489, 0.0258, and 0.0086, respectively. Median NDVI was negative (−0.311), while median NDWI and MNDWI were positive (0.412 and 0.761). This ordering is consistent with the intended water-like design, but it does not establish equivalence to real atmospherically corrected satellite reflectance (see Table 8 and Table 9).

### 5.4. Reconstruction Performance on the Shared Cohort

**Figure 4:**
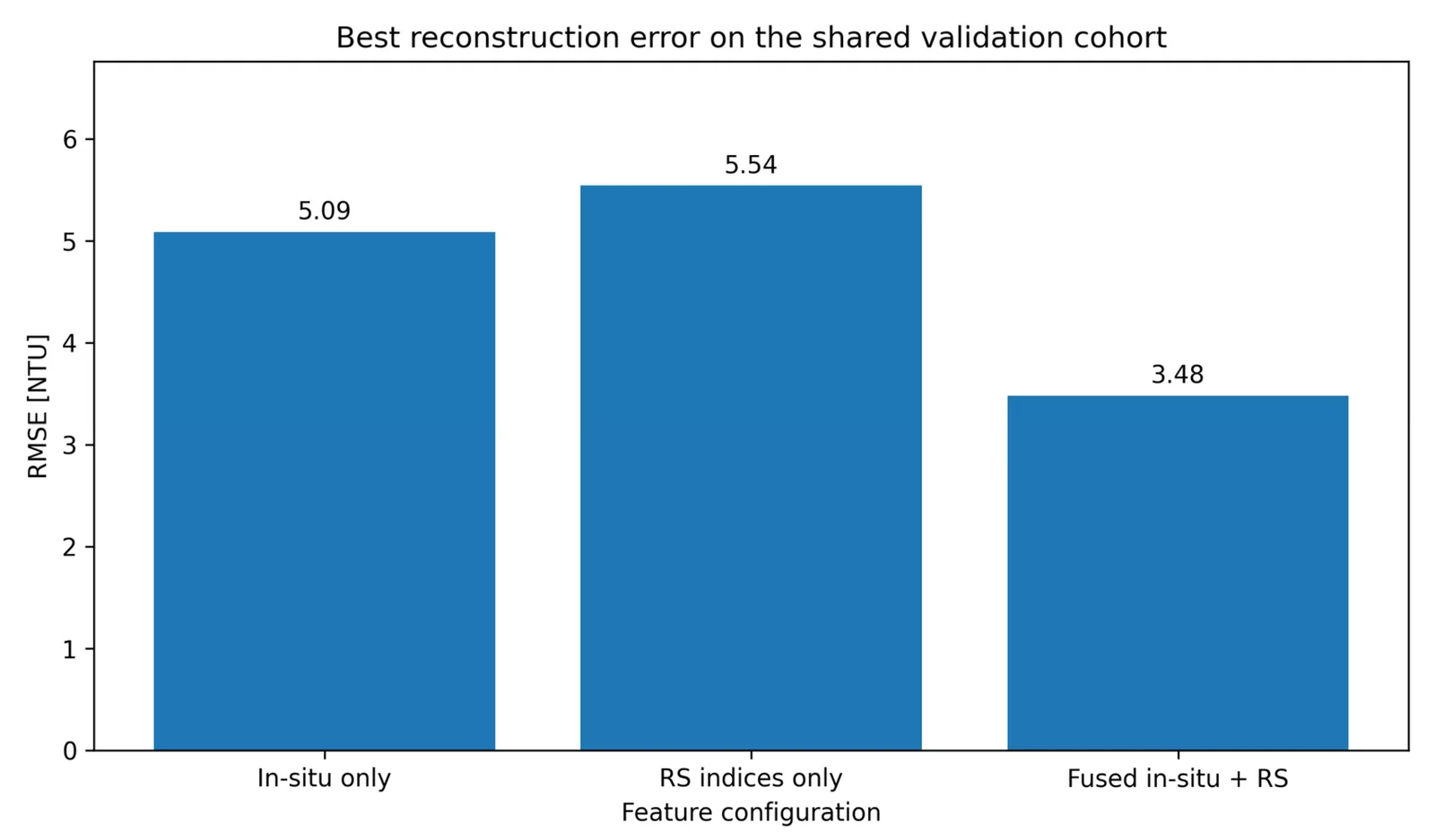
Minimum validation RMSE for each feature configuration. All bars are based on the same 667 training and 287 validation records.

Ridge regression was the best method for all three configurations. The fused configuration achieved RMSE = 3.481 NTU, MAE = 2.768 NTU, and R^2^ = 0.729. The in situ-only configuration achieved RMSE = 5.086 NTU and R^2^ = 0.421, whereas the spectral-index-only configuration achieved RMSE = 5.538 NTU and R^2^ = 0.313 (see Table 10 and Figure 4).

### 5.5. Observed Versus Predicted Synthetic Turbidity

**Figure 5:**
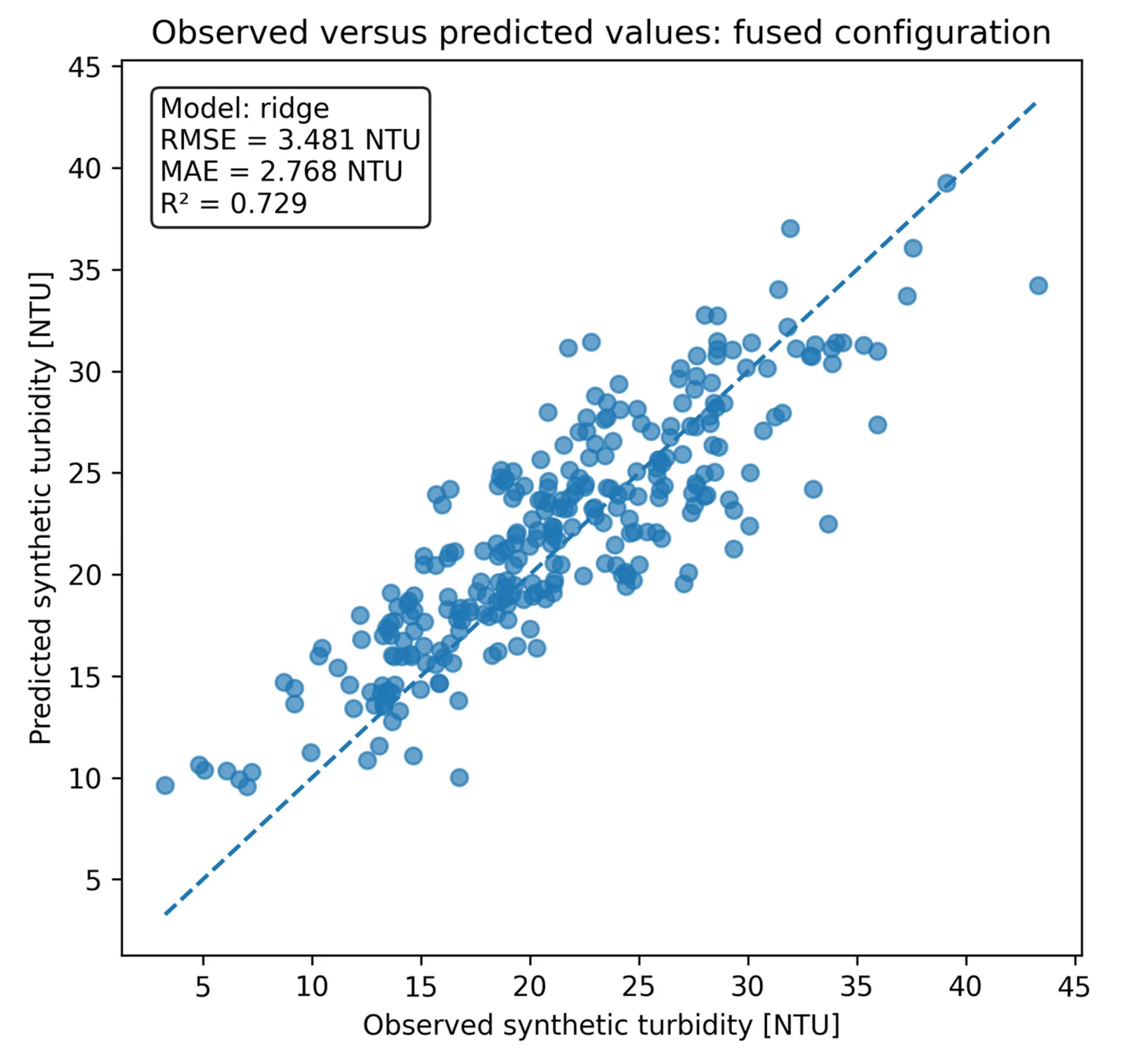
Observed versus predicted synthetic turbidity for the best fused model (ridge regression). RMSE = 3.481 NTU, MAE = 2.768 NTU, and R^2^ = 0.729 on 287 validation records.

The fused model reproduced the overall gradient of synthetic turbidity across the validation period. Residual dispersion remained visible, as expected from the target noise and imperfect temporal correspondence. Figure 5 reports the model and all principal performance metrics directly to avoid requiring cross-reference to a separate table.

### 5.6. Reproducibility Checks

The complete benchmark was rerun from the configuration file, and the generated counts, shared cohort, metrics, tables, and figures were reproduced deterministically. The repository test suite validates configuration loading, data-layer operations, spectral indices, temporal alignment, feature generation, model benchmarking, the prepared-data training pipeline, and the complete fusion benchmark. The test suite contains 33 tests; 32 pass in the base environment and one optional-dependency test is skipped when the corresponding external package is unavailable.

## 6. Discussion

### 6.1. Meaning of the Fusion Result

The fused configuration outperformed both single-source configurations because the benchmark was deliberately constructed around complementary information. In situ variables represented meteorological, physicochemical, seasonal, and station contexts, while spectral indices indirectly represented the latent suspended-matter process. The result therefore verifies that AI-Aquatica-RS can preserve and combine complementary signals through its alignment and modeling workflow. It does not demonstrate that fusion will improve every real water-quality application.

The strongest methodological correction relative to the initial experiment is the use of one shared matched cohort. Comparing 1080 in situ-only records with 954 fused records would confound feature-source effects with sample-size and split differences. Restricting all configurations to the same 954 records and the same chronological split makes the benchmark internally comparable and directly addresses this source of bias.

### 6.2. Unique Acquisitions Versus Aligned Modeling Records

The distinction between 181 acquisitions and 954 modeling records is essential. The former is the number of unique remote-sensing-like observations and is the correct denominator for acquisition-level spectral summaries. The latter is the number of daily in situ observations that found a valid nearest acquisition within ±3 days. One acquisition can be the nearest match for multiple in situ dates. Reporting both levels prevents duplicated aligned values from being mistaken for independent satellite acquisitions.

### 6.3. Temporal Tolerance as an Explicit Modeling Decision

Increasing tolerance raised coverage from 16.76% for exact-date matching to 88.33% at ±3 days. This gain comes with a temporal-fidelity cost: the mean absolute difference increased from zero to 1.31 days. The appropriate window in a real study depends on variable dynamics, hydrological events, satellite revisit, cloud frequency, and sampling design. Rapidly changing variables may require stricter matching or event-aware filtering, whereas slowly varying properties may tolerate wider windows [17,39,40,41]. AI-Aquatica-RS exposes the tolerance as a configuration parameter and exports its coverage consequences.

### 6.4. Synthetic Reflectance Realism and Limitations

The generator corrects the previous high-NIR behavior by constraining NIR and SWIR below the visible bands for most scenarios. The resulting negative NDVI and positive water indices are qualitatively water-like. Nevertheless, the values are only reflectance proxies. They do not include sensor spectral response, atmospheric correction, bidirectional effects, adjacency contamination, bottom reflectance, or pixel mixing. The scenario comparison in Figure 2 is therefore a generator diagnostic, not a validation against Landsat or Sentinel-2.

Real-data validation must compare extracted satellite reflectance with coincident field measurements and should document product level, correction algorithm, cloud mask, spatial footprint, and water type [15,16,17,18,38]. It should also include spatially blocked or leave-one-station-out validation to account for autocorrelation and site dependence. These requirements are beyond the present controlled software benchmark.

### 6.5. Software Contribution and Reuse

The principal contribution is a reproducible integration layer. The configuration file records experimental choices; traceability fields document each match; the common-cohort rule supports fair comparison; and machine-readable outputs allow figures and tables to be regenerated without manual transcription. Example CSV files and the output manifest make the practical structure visible to readers and potential users.

The modular design permits extension with sensor-specific band mappings, additional spectral indices, uncertainty estimation, alternative matching rules, spatial validation, time-series models, and connectors to geospatial preprocessing systems. The same framework can also be used with real tables after upstream satellite processing, provided the resulting claims are validated against appropriate observations.

## 7. Software Availability and Reuse

AI-Aquatica-RS is distributed as a Python 3.11 package with source code, tests, example configurations, documentation, and generated benchmark outputs. The release adds CITATION.cff, a complete article_benchmark.yaml configuration, a run-fusion-benchmark command, shared-cohort validation tests, example input and aligned-output files, descriptive-statistics exports, an output manifest, and a human-readable benchmark specification.

The project targets Python 3.11 or later and uses NumPy 1.26, pandas 2.1, PyArrow 14.0, Pydantic 2.6, PyYAML 6.0, scikit-learn 6.0, and Matplotlib 3.11. Optional geospatial and external boosting dependencies are isolated from the base workflow. Continuous-integration configuration and deterministic tests support future maintenance.

## 8. Conclusions

This study presented AI-Aquatica-RS, a modular Python framework for reproducible fusion of remote-sensing-derived spectral indices and in situ water-quality observations. The software makes temporal matching, feature construction, model comparison, and result export explicit and repeatable.

A fully synthetic controlled benchmark generated 1080 in situ-like observations and 181 unique remote-sensing-like acquisitions. A ±3-day tolerance produced a common matched cohort of 954 records. When all feature configurations were evaluated on the same 667 training and 287 validation records, the fused ridge model achieved RMSE = 3.481 NTU, MAE = 2.768 NTU, and R^2^ = 0.729, outperforming both single-source configurations.

These results demonstrate the correct recovery of designed complementarity and reproducible end-to-end execution. They do not constitute validation on real aquatic observations. Future work should apply the framework to sensor-specific, atmospherically corrected satellite products and independently collected field measurements using spatially and temporally robust validation protocols.

## Figures and Tables

**Table 1:** Principal software layers and responsibilities.

Layer	Responsibility
Data input	CSV and Parquet loading, required-column checks, datetime parsing, and station-key validation.
Spectral indices	Calculation of NDVI, NDWI, MNDWI, and NDTI from red, green, NIR, and SWIR columns.
Temporal fusion	Exact or nearest matching within a user-defined tolerance, with matched row identifier, matched acquisition date, match type, and signed time difference.
Feature engineering	Calendar variables, cyclical day-of-year encoding, station indicators, and alternative single-source or fused feature matrices.
Reconstruction	Deterministic benchmarks covering central-tendency baselines, linear models, k-nearest neighbors, decision trees, random forests, extra trees, and gradient boosting.
Result export	CSV summaries, predictions, example records, output manifest, benchmark specification, and publication-ready figures.

**Table 2:** Controlled benchmark generation parameters.

Component	Generation Rule
Stations and period	6 stations; 180 daily observations per station; 1 January–28 June 2024.
Rainfall	Zero with probability 0.69; otherwise Gamma (shape = 1.25, scale = 3.2) mm.
Antecedent rainfall	Previous proxy multiplied by 0.62 plus current rainfall.
Water temperature	13.5 + 7.8 × seasonal component + Gaussian noise SD 0.85 °C.
Conductivity	475 + station increment + 120-day cyclic term − 1.4 × antecedent rainfall + Gaussian noise SD 6.5 µS/cm.
pH	7.52 + seasonal and latent algal components + Gaussian noise SD 0.035.
RS acquisition process	Nominal five-day revisit; independent missing-acquisition probability 0.18.
Random state	42 for environmental variables; independent deterministic stream for acquisition availability.

**Table 3:** Example synthetic in situ-like input records.

Station	Date	Temp. (°C)	Cond. (µS/cm)	pH	Rain (mm)	Turbidity (NTU)
station-1	2024-01-01	12.75	495.86	7.503	0.00	10.98
station-1	2024-01-02	13.82	498.24	7.563	0.00	9.53
station-1	2024-01-03	13.77	488.02	7.567	0.00	9.09
station-1	2024-01-04	14.56	497.99	7.527	0.00	10.57

**Table 4:** Example synthetic remote-sensing-like acquisitions.

Station	Date	Scenario	Green	Red	NIR	SWIR	NDTI
station-1	2024-01-01	clear	0.0485	0.0318	0.0162	0.0047	−0.208
station-1	2024-01-06	clear	0.0494	0.0330	0.0179	0.0062	−0.199
station-1	2024-01-16	clear	0.0561	0.0466	0.0220	0.0071	−0.093
station-1	2024-01-21	turbid	0.0641	0.0618	0.0274	0.0094	−0.018

**Table 5:** Principal exported benchmark files.

Output File	Description
metrics_by_feature_set.csv	Metrics for every model and feature configuration
best_models_by_feature_set.csv	Best model for each shared-cohort configuration
predictions_best_models.csv	Validation predictions for the best models
temporal_alignment_coverage.csv	Coverage for each temporal tolerance
modeling_cohort_summary.csv	Unique acquisition and common-cohort counts
in_situ_variables_summary.csv	Descriptive statistics for synthesized in situ variables
spectral_bands_summary_acquisition_level.csv	Acquisition-level band statistics
spectral_indices_summary_acquisition_level.csv	Acquisition-level spectral-index statistics
sample_aligned_modeling_cohort.csv	Example aligned modeling records
BENCHMARK_SPECIFICATION.md	Human-readable benchmark definition

**Table 6:** Descriptive statistics for synthesized in situ-like variables.

Variable	n	Mean	SD	Min	Median	Max
Water temperature (°C)	1080	15.978	4.969	3.642	17.490	23.654
Conductivity (µS/cm)	1080	550.540	55.851	435.647	551.477	662.231
pH	1080	7.599	0.103	7.320	7.623	7.790
Rainfall (mm/24 h)	1080	1.182	2.531	0.000	0.000	19.116
Turbidity (NTU)	1080	22.002	7.107	3.160	21.740	45.598

**Table 7:** Temporal alignment coverage.

Tolerance (Days)	In Situ Rows	Unique RS Acquisitions	Matched Rows	Coverage (%)	Mean |Δt| (Days)
0	1080	181	181	16.76	0.00
1	1080	181	538	49.81	0.66
3	1080	181	954	88.33	1.31
5	1080	181	1047	96.94	1.58
7	1080	181	1067	98.80	1.67

**Table 8:** Acquisition-level descriptive statistics for synthetic bands and spectral indices.

Variable	n	Mean	SD	Min	Median	Max
GREEN	181	0.0619	0.0076	0.0448	0.0617	0.0831
RED	181	0.0489	0.0112	0.0259	0.0472	0.0869
NIR	181	0.0258	0.0052	0.0125	0.0253	0.0454
SWIR	181	0.0086	0.0022	0.0038	0.0084	0.0170
NDVI	181	−0.3067	0.0514	−0.4494	−0.3110	−0.1356
NDWI	181	0.4166	0.0475	0.2864	0.4124	0.5643
MNDWI	181	0.7588	0.0372	0.6606	0.7608	0.8732
NDTI	181	−0.1257	0.0807	−0.3730	−0.1290	0.0625

**Table 9:** Qualitative design check against typical satellite-derived water behavior.

Diagnostic	Synthetic Result	Typical Water-Like Expectation	Interpretation
NIR relative to green	0.0258 versus 0.0619	Water pixels generally show reduced NIR relative to visible reflectance [4,17,36].	Qualitatively consistent
SWIR relative to red	0.0086 versus 0.0489	Water strongly absorbs SWIR, which is typically below visible reflectance [13,17].	Qualitatively consistent
NDVI	Median −0.311	Open-water pixels are commonly negative when NIR is lower than red [29,36].	Qualitatively consistent
NDWI	Median 0.412	Water-enhancement formulations are generally positive when green exceeds NIR [13,31].	Qualitatively consistent
Sensor equivalence	Not evaluated	Numerical comparison requires a specified sensor, product level, atmospheric correction, and coincident scene.	No equivalence claimed

**Table 10:** Best model for each feature configuration using the identical shared cohort and temporal split.

Feature Configuration	Best Model	Train	Validation	RMSE (NTU)	MAE (NTU)	R^2^	MAPE (%)
In situ only	Ridge	667	287	5.086	3.869	0.421	20.67
RS indices only	Ridge	667	287	5.538	4.663	0.313	26.39
Fused in situ + RS	Ridge	667	287	3.481	2.768	0.729	15.55

## Data Availability

The source code, configuration-driven controlled benchmark, generated synthetic inputs, model outputs, and representative exported files are publicly available in the GitHub repository: https://github.com/TyMill/AI-Aquatica-RS v1.0.0 (accessed on 1 June 2026). The archived release is available on Zenodo under DOI: https://doi.org/10.5281/zenodo.20547995 (accessed on 1 June 2026). All benchmark observations are synthetic and were generated by the software; they are not derived from real satellite scenes or field-monitoring observations.
